# A Case Report of Clonazepam Dependence: Utilization of Therapeutic Drug Monitoring During Withdrawal Period

**DOI:** 10.1097/MD.0000000000002881

**Published:** 2016-03-07

**Authors:** Ivana Kacirova, Milan Grundmann, Petr Silhan, Hana Brozmanova

**Affiliations:** From the Department of Clinical Pharmacology (IK, MG, HB), Faculty of Medicine, University of Ostrava; Department of Clinical Pharmacology (IK, HB), Department of Laboratory Diagnostics; and Department of Psychiatry (PS), University Hospital Ostrava, Czech Republic.

## Abstract

Clonazepam is long-acting benzodiazepine agonist used in short-acting benzodiazepine withdrawal; however, recent observations suggest the existence of its abuse.

We demonstrate a 40-year-old man with a 20-year history of psychiatric care with recently benzodiazepine dependence (daily intake of ∼60 mg of clonazepam and 10 mg of alprazolam). High serum levels of both drugs were analyzed 3 weeks before admission to hospitalization (clonazepam 543.9 ng/mL, alprazolam 110 ng/mL) and at the time of admission (clonazepam 286.2 ng/mL, alprazolam 140 ng/mL) without any signs of benzodiazepine intoxication. Gradual withdrawal of clonazepam with monitoring of its serum levels and increase of gabapentin dose were used to minimize physical signs and symptoms of clonazepam withdrawal. Alprazolam was discontinued promptly. Clinical consequences of the treatment were controllable tension, intermittent headache, and rarely insomia.

It is the first case report showing utilization of therapeutic drug monitoring during withdrawal period in the patient with extreme toleration to severe benzodiazepine dependence.

## INTRODUCTION

Humans have been using hypnotic-sedative agents for many centuries. Barbiturates appeared in the 1930s, and the first benzodiazepine (chlordiazepoxide) was marketed in the early 1960s. Benzodiazepines are among the most commonly prescribed psychotropic medications worldwide and the prevalence of long-term use in the general population is ∼2% to 7%. The problem of use of benzodiazepines is related to the use of high doses and associated with the use of the drugs have been abuse, dependence, and withdrawal sequelae.^1,2^ Clonazepam as observed with other benzodiazepines has hypnotic, sedative, anxiolytic, anticonvulsant, muscle relaxant, and amnesic properties. It is rapidly and completely absorbed after oral administration and extensively metabolized in the liver, primarily by cytochrome P450 (CYP) isoenzymes 3A4, to its major metabolite 7-aminoclonazepam which has weak anticonvulsant activity and is excreted mainly in urine.^3^ CYP3A4 is known to be involved in the metabolism of a wide variety of xenobiotics and has a large potential for drug interactions. This enzyme is also documented to exhibit a genetic polymorphism.^4^ Clonazepam pharmacokinetic parameters are presented in Table 1.^5–7^ Potential problems associated with improper use or abuse of clonazepam include physical and psychological dependence, suicidal thoughts or actions, worsening of depression, sleep disorders, and aggression.^8^ Central nervous system depression and rarely cardiorespiratory depression characterize oral benzodiazepine overdoses. Cyclic coma in a 4-year-old boy followed by an ingestion of clonazepam was reported with a plasma level of 69 ng/mL on blood drawn shortly after admission. The level in the 17 kg patient would be equivalent to 14 to 32 mg in an adult. That means, an acute clonazepam overdose of 14 to 32 mg taken orally produced a plasma concentration of 69 ng/mL.^9^ At clonazepam plasma concentrations >100 ng/mL additionally reflected in therapeutic doses, toxic symptoms such as drowsiness and ataxia can occur.^10^ A table of toxicological drug concentrations compiled by Regenthal et al^11^ indicates that 1000 ng/mL clonazepam is a comatose-lethal concentration. A case report of a fatal drug interaction caused by ingestion of oxycodone and clonazepam was reported by Burrows et al.^12^ Quantitative analysis of the femoral plasma revealed a clonazepam concentration of 1410 ng/mL. The reported concentration reflected the concentration present at the time of death and was not falsely elevated due to postmortem redistribution as a femoral specimen was used for analysis.^12^ Some published studies have reported the use of clonazepam in benzodiazepine withdrawal; however, recent observations suggest the existence of clonazepam abuse.^13^ A case report of megadose clonazepam dependence was reported by Mowla et al.^14^ A 24-year-old woman was using ∼180 mg/day of clonazepam in 3 divided doses without any medical problem. The patient was able to tolerate such a high dose of clonazepam without evident impairment in psychomotor function, speech, orientation, and consciousness. She only experienced uncontrollable tension and insomnia after tapering down clonazepam. However, serum concentrations of clonazepam were not stated.^14^ Because tolerance to clonazepam develops in many patients, it has been difficult to identify a clear correlation between serum levels of clonazepam and either efficacy or toxicity. In patients with epilepsy treated with therapeutic doses of clonazepam, serum concentrations in the order of 20 to 70 ng/mL have been reported and drug concentrations above the recommended reference range that causes the laboratory to feedback immediately to the prescribing physician (i.e., laboratory alert level) is 80 ng/mL.^15,16^ The therapeutic reference range/recommended drug concentration of clonazepam used as anxiolytic/hypnotic drugs is 4 to 80 ng/mL (the “laboratory alert level” is 100 ng/mL).^16^ Alprazolam is a triazolobenzodiazepine used in the treatment of anxiety, depression, and panic attacks. It is subject to abuse and abusers are more likely to be men and often adolescent.^17^ The drug is the intermediate to short-acting benzodiazepine with elimination half-life between 9 and 16 h. It is metabolized by CYP3A4/5, yielding alpha-hydroxy- and 4-hydroxy-alprazolam as principal initial metabolites. Both have lower intrinsic benzodiazepine receptor affinity than alprazolam and appear in human plasma at <10% of the concentrations of the parent drug. Side effects (drowsiness, sedation, etc.) are consistent with its primary benzodiazepine agonist action and increase in frequency with higher steady-state plasma concentrations. As with other benzodiazepines, tolerance develops to the central depressant effects of alprazolam and side effects diminish also over time with continuous administration. Because of its relatively short half-life, abrupt termination of alprazolam treatment can be followed by 1 or more discontinuation syndromes (recurrence, rebound, or withdrawal). The CNS depressant effects became markedly more prevalent once the concentration rose >60 to 70 ng/mL. At any given daily dosage, actual steady-state plasma concentration varies considerably among patients. These differences are attributable to variations in metabolic clearance and possibly also to incomplete compliance with the prescribed dosage regimen.^18,19^ For example, plasma levels of the patients taking a daily dose of 6 mg ranged from 40 to 107 ng/mL,^20^ and the wide distribution of steady-state plasma alprazolam concentrations among 94 patients receiving a daily dose of 5 mg/day was also found (range = 0–181 ng/mL).^19^ In the study of Greenblatt et al,^21^ the mean daily dose was 5.7 ± 2.3 mg/day (33% of patients were receiving daily doses in the range of 7 to 10 mg/day) and the mean plasma alprazolam concentration was 60 ± 40 ng/mL, with a range of 0 to 214 ng/mL.^21^ Currently, the therapeutic reference range/recommended drug concentration of alprazolam is 5 to 50 ng/mL (“laboratory alert level” is 100 ng/mL). In chronic users, effective plasma concentrations can be markedly higher than in nonusers.^16^ To date, there is no specific or internationally recognized treatment for dependence on benzodiazepines. Although the types of intervention differ, the common aim of treatment continues to be total abstinence from the drugs. However, most patients suffering from high-dose dependence fail to achieve long-term abstinence, and in such cases some clinicians have been using benzodiazepine “substitution” treatment for decades.^22,23^ Therapeutic drug monitoring (TDM) is the specific method of clinical pharmacology for the monitoring of therapy by using measurement of drug serum concentrations followed by interpretation and good cooperation with clinician. It is a powerful tool that allows tailoring the treatment to the specific needs of individual patients. TDM can help in personalized medicine in monitoring adherence, dose adjustment, minimalization of side effects, decrease of mortality and morbidity, and reduction of cost of health care also in the treatment of psychiatric disorders. Phenotyping and genotyping can increase therapeutic drug monitoring on higher level.^24–26^ In the case report, we would like to demonstrate a utilization of TDM during withdrawal period in a patient with severe benzodiazepine dependence.

**TABLE 1:**
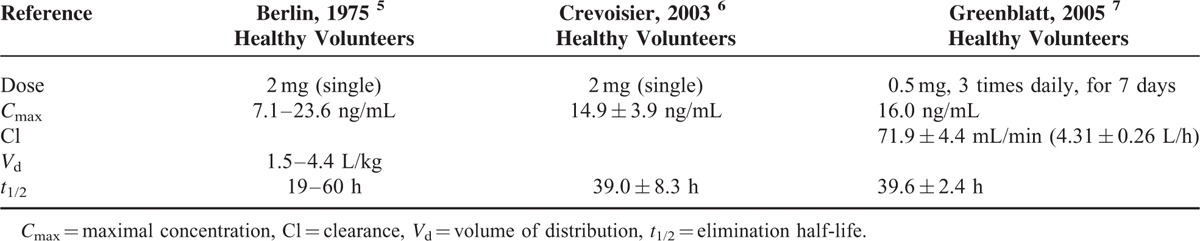
Pharmacokinetics of Clonazepam

## METHODS

A 40-year-old man, unmarried and childless, was voluntarily admitted to the Department of Psychiatry of University Hospital to detoxification with recommendation of outpatient psychiatrist. The patient with a 20-year history of psychiatric care started during military service has been treated for schizophrenia, schizoid disorder, social phobia, anxiety disorder, alcohol abuse, and recently for benzodiazepine dependence. Furthermore he suffered from hypertension, hyperbilirubinemia, and obesity (weight 110 kg, height 175 cm, BMI 35.6) and was nonsmoker. His brother (twin) has been treated for bipolar disorder and his grandfather suffered also from psychiatric disorder. The patient was unemployed and received partial disability annuity. The attending outpatient psychiatrist appealed for careful and gradual reducing of benzodiazepine because the patient mentioned strong anxiety and panic attack during previous quick and rapid attempt to treat benzodiazepine dependence with failure of treatment. The patient reported at admission daily intake of ∼60 mg of clonazepam and 10 mg of alprazolam. High serum levels of both drugs were analyzed 3 weeks before admission to hospitalization (clonazepam 543.9 ng/mL, alprazolam 110 ng/mL) and at the time of admission (clonazepam 286.2 ng/mL, alprazolam 140 ng/mL). The patient showed symptoms of disinhibition, especially impulsivity and aggressive outbursts. However, any other signs of benzodiazepine intoxication including amnesia, fall, gait dysfunction, ataxia, or epileptic seizures were not found and nothing abnormal in routine laboratory data including hepatic enzymes was analyzed with exception of higher serum level of bilirubin (Gilbert syndrome). Moreover, he used gabapentin 600 mg 3 times daily, citalopram 20 mg once a day, sulpirid 50 mg 1 to 3 times daily and betaxolol 10 mg once a day. The patient planned partial detoxification during hospitalization with continuation in psychiatric sanatorium. The number and type of both subjective and physical withdrawal signs and symptoms were recorded in the patient's chart. Quantitative analysis for clonazepam was performed by high-performance liquid chromatography (HPLC) method.^27^ Trough (i.e. before administration) blood samples of clonazepam were collected consecutively 8 times during withdrawal period. Pharmacokinetic analysis of every clonazepam level was performed using software MWPharm version 3.30 and interpreted by the clinical pharmacologist. The therapeutic reference range of clonazepam used in our department was 20 to 80 ng/mL. The alpazolam level was analyzed by the HPLC method by Mucha et al.^28^ The patient signed Informed Consent Form at the time of admission to hospitalization. An approval of ethics committee was not necessary because of the routine health care.

## RESULTS

**FIGURE 1:**
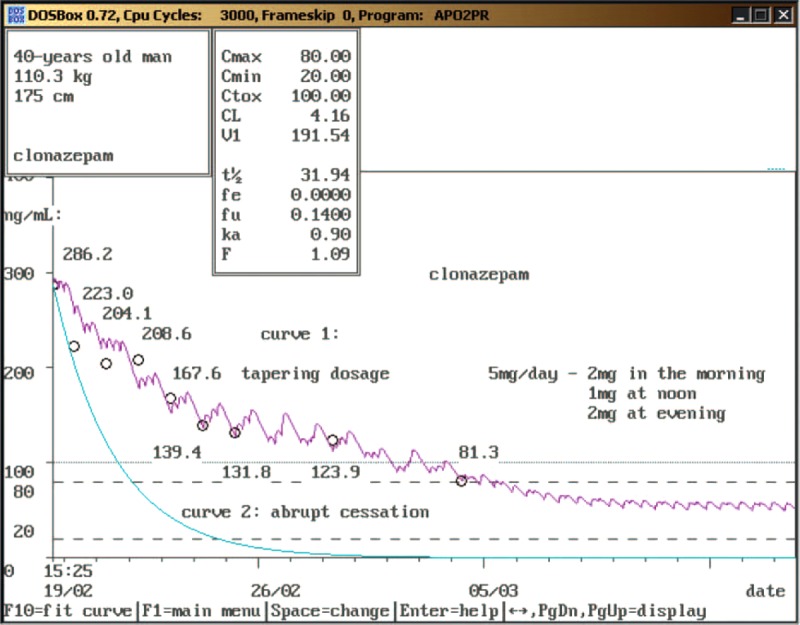
Pharmacokinetics analysis of clonazepam serum levels during the tapering period (curve 1) and hypotetical abrupt cessation (curve 2) (color in print is not required).

The patient used clonazepam 4 mg 3 times daily first 8 days after admission and then the dosage was wind down using a rate of taper off by ∼2 mg/2–3 days with almost daily therapeutic monitoring of its level. Changes in clonazepam concentrations and pharmacokinetic parameters during gradual withdrawal period are summarized in Table 2. The clearance was decreased from the 1st to the 4th day of hospitalization and then remained similar to healthy volunteers.^7^ The elimination half-life was increased to the 4th day and then was stable similar to general population.^5–7^Figure 1 shows differences between serum levels of clonazepam during tapering dosage (when the serum level in the therapeutic range was achieved in ∼2 weeks) and within hypotetical abrupt cessation (when the upper limit of therapeutic range should be reached in 2 days and a nondetectable serum level in 1 week). The patient only suffered from controllable tension and headache namely at the beginning of hospitalization and rarely insomnia at the end. He has not experienced any other withdrawal symptoms (such as tremor, irritability, sweating, hallucinations, aggressiveness) during hospitalization, but his weight decreased by ∼3.6 kg. However, 1 mg of alprazolam was administrated 2 times early after admission in the case of anxiety and/or tension and then alprazolam was discontinued. A total of 400 mg of ibuprofen (1 or 2 times daily) was administered at headache and 10 mg of zolpidem in the case of insomnia. The dosage of gabapentin was increased to 900 mg 3 times daily (as prevention of epileptic seizures during reduction of benzodiazepines) and the dosage of citalopram and betaxolol was not changed. The blood pressure was measured 3 times daily with the values 110–120/70–80 mm Hg. The patient used clonazepam 5 mg daily (2 mg in the morning, 1 mg at noon, and 2 mg at evening), gabapentin 900 mg 3 times daily, citalopram 20 mg once a day, and betaxolol 10 mg once a day at the end of hospitalization. He was discharged 16th day with the dose of clonazepam 5 mg daily (2 mg in the morning, 1 mg at noon, and 2 mg at evening), the clonazepam serum level 81.3 ng/mL (i.e. in upper limit of therapeutic range) and without clinical signs of withdrawal. Admission in psychiatric sanatorium to detoxification was planned at the same day.

**TABLE 2:**
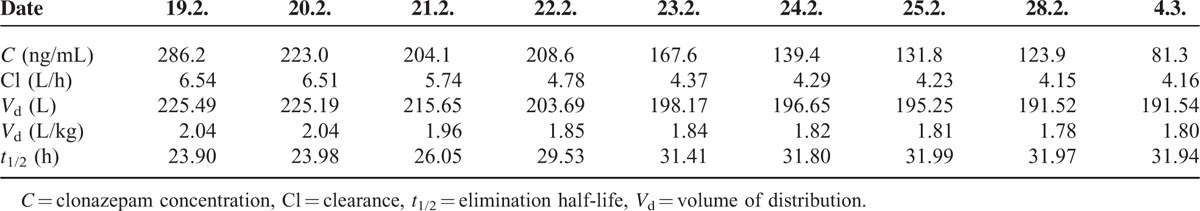
Changes in Clonazepam Pharmacokinetics of the Patient During Gradual Withdrawal Period

## DISCUSSION

The keystones of management of benzodiazepine withdrawal are slow tapering of the dose and psychological support for the patient, when necessary. Benzodiazepines should not be withdrawn abruptly because there is a risk of epileptic fits or of the patient becoming confused or experiencing paranoid psychosis. Nevertheless, abrupt cessation can be justified if a very serious adverse effect supervenes during treatment.^29^ However, severe withdrawal symptoms did not occur in our case report despite of relatively short half-life alprazolam discontinuation because the patient continued to use long-acting, high-potency benzodiazepine agonist clonazepam. Gradual withdrawal of clonazepam with almost daily monitoring of its serum levels (Figure 1) and increase of gabapentin dose were used to minimize physical signs and symptoms of clonazepam withdrawal. Clinical consequences of the treatment were only controllable tension, intermittent headache, and rarely insomnia. Recent observations suggest the existence of clonazepam abuse;^13^ however, only the case report of megadose clonazepam dependence was reported by Mowla et al^14^ without monitoring of clonazepam plasma levels. To our knowledge, there is no published record of such high serum levels of clonazepam (543.9 ng/mL) without any signs of benzodiazepine intoxication. This could be explained by changes in γ-aminobutyric acid (GABA) receptors. GABA is the main inhibitory neurotransmitter in humans. It exerts the majority of its effects through ionotropic GABA_A_ receptors. Benzodiazepines bind to a modulatory site on the GABA_A_ receptor complex, enhancing (agonist action) GABA_A_ activity. Chronic high dose use of benzodiazepines causes allosteric alterations of the sites for benzodiazepines reducing the affinity between the site for benzodiazepines and site for GABA in the GABA_A_ complex.^2,30,31^ It seems important to follow clonazepam use because its use has increased and the increase in the drug availability can be a factor influencing its misuse.^13,32^ Discontinuation of existing benzodiazepine usage is a desirable goal, but it can be difficult.^29^ Therapeutic monitoring of alprazolam and especially clonazepam can help to discontinue these drugs without severe withdrawal symptoms. In conclusion, benzodiazepines are among the most commonly prescribed psychotropic medications worldwide. The risk of dependence after long-term use of benzodiazepines has been described, as reflected in the appearance of a series of symptoms when the drug is abruptly withdrawn.^2^ In our case report we would like to introduce clonazepam and alprazolam dependence liability in “real life.” Alprazolam was discontinued almost promptly without severe withdrawal symptoms because the patient continued to use long-acting, high-potency benzodiazepine agonist clonazepam. Gradual withdrawal of clonazepam with almost daily monitoring of its serum levels and increase of gabapentin dose were used to minimize physical signs and symptoms of clonazepam withdrawal. It is, to our knowledge, the first case report showing utilization of TDM during withdrawal period in the patient with extreme toleration to severe benzodiazepine dependence.
